# Marine Metagenome as A Resource for Novel Enzymes

**DOI:** 10.1016/j.gpb.2015.10.001

**Published:** 2015-11-10

**Authors:** Amani D. Alma’abadi, Takashi Gojobori, Katsuhiko Mineta

**Affiliations:** Computational Bioscience Research Center (CBRC), King Abdullah University of Science and Technology (KAUST), Thuwal 23955-6900, Saudi Arabia

**Keywords:** Microbial diversity, Culture-independent studies, Catalysis, Lipase, Biotechnology

## Abstract

More than 99% of identified prokaryotes, including many from the marine environment, cannot be cultured in the laboratory. This lack of capability restricts our knowledge of microbial genetics and community ecology. Metagenomics, the culture-independent cloning of environmental DNAs that are isolated directly from an environmental sample, has already provided a wealth of information about the uncultured microbial world. It has also facilitated the discovery of novel biocatalysts by allowing researchers to probe directly into a huge diversity of enzymes within natural microbial communities. Recent advances in these studies have led to a great interest in recruiting microbial enzymes for the development of environmentally-friendly industry. Although the metagenomics approach has many limitations, it is expected to provide not only scientific insights but also economic benefits, especially in industry. This review highlights the importance of metagenomics in mining microbial **lipases**, as an example, by using high-throughput techniques. In addition, we discuss challenges in the metagenomics as an important part of bioinformatics analysis in big data.

## Introduction

Recent developments in catalysis have led to a renewed interest in the use of enzymes for the environmentally-friendly industry. Most industrially-relevant enzymes are of microbial origin [1]. Identification and isolation of microbial enzymes are thus important steps in improving industrial processes, although only less than 1% of environmental bacteria can be cultivated in the laboratory [2], [3], [4].

**Figure 1:**
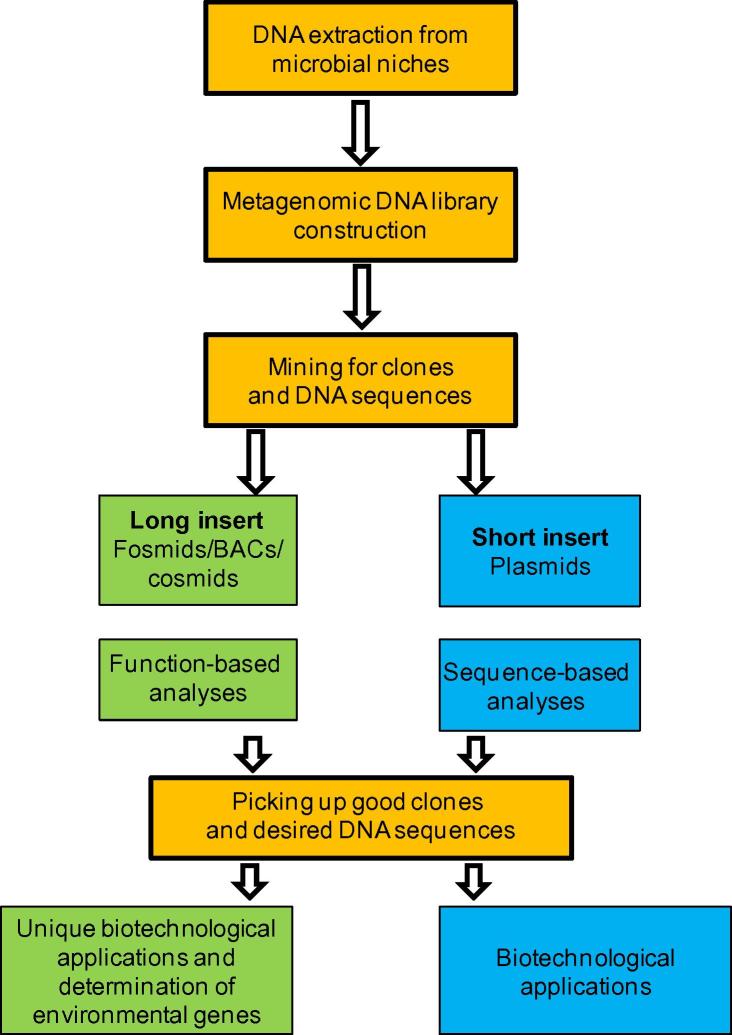
**The process of functional metagenomics of marine microbes from environmental samples** This flowchart illustrates how metagenome is analyzed with the emphasis on the four important processes. BAC, bacterial artificial chromosome.

The current challenging questions have arisen regarding the discovery, identification, and function validation of the uncultured microorganisms. Metagenomics study, which usually starts from the isolation of environmental DNAs without culture, has emerged as an excellent means to study biodiversity and biotechnological applications in certain conditions such as marine environments (Figure 1) [5], [6], [7]. It provides insights into the genomic pool of microorganisms that are recovered directly from environmental sources. Thus, metagenomics can be used for not only exploring ecological and environmental puzzles, but also finding unique biocatalysts with promising characteristics for biotechnological applications [8], [9], [10]. In particular of the biotechnological applications, metagenome libraries could be screened based on either protein function or nucleotide sequences.

Function-based screening is a direct way of identifying novel enzymes [2]. In this method, enzyme activities are assayed by harvesting a metagenomic library on agar plates enriched with substrates. Positive clones may then be recognized by visual screening for a clear zone called a halo [11]. As a result, function-based screening selects clones with functional activities, such as the synthetic and degradation activities. Unlike sequence-based approaches mentioned later, functional-based screening does not require identification of homologies to genes of known functions. It therefore contributes to nucleic acid and protein databases by adding novel functional annotations. However, this method often suffers from a number of limitations, such as a low hit rate of positive clones, low throughput, and time-consuming screening [11].

In contrast, in sequence-based screening, which involves metagenomic DNA sequencing using next-generation sequencing (NGS) technology, microbial enzymes and bioactive compounds can be explored from niches of interest [10]. However, sequence-based screening requires the detection of gene variants with conserved domain or motif of the known functions for enzymes identifications. This approach does not necessarily identify the novel genes.

In light of an increasing demand for enzymes such as carbohydrases, proteases, polymerases, nucleases, and lipases, it is becoming extremely difficult to ignore the importance of hydrolytic enzymes as potential biocatalysts in a wide variety of industries, including chemical processing, dairy, agrochemicals, paper, cosmetics, pharmaceuticals, surfactants, detergents, polymers, and biofuel synthesis [12], [13]. For example, a lipase is often used at the consumable detergent, as it can hydrolyze fat from clothes and thus enhance its cleaning efficiency. Therefore, the hydrolytic enzymes have been used as promising environmentally-friendly biocatalysts in various industries.

According to the Nomenclature Committee of the International Union of Biochemistry and Molecular Biology, enzymes are classified into six main classes (Table 1). One of the most important classes is hydrolases (E.C.3.-.-.-), which catalyze the hydrolytic cleavage of different types of chemical bonds. Many commercially-critical enzymes belong to this class, *e.g.*, proteases, amylases, acylases, lipases, and esterases [14]. Lipases are simply hydrolytic enzymes that catalyze hydrolysis and synthesis reactions by breaking down triacylglycerides into free fatty acids and glycerols, which act under aqueous conditions on the carboxyl ester bonds present in triacylglycerols to liberate fatty acids and glycerol [15], [16], [17]. Hydrolysis of glycerol esters carrying an acyl chain, which comprises less than 10 carbon atoms in length, with tributyrylglycerol (tributyrin) as the standard substrate, usually indicates the presence of an esterase. Most lipases are able to hydrolyze esterase substrates [18]. These reactions usually continue with high regio- and/or enantio-selectivity, making lipases a valuable group of biocatalysts in organic chemistry [19], [20], [21].

Two criteria are used to determine if a lipolytic enzyme is a genuine lipase (EC.3.1.1.3) (Table 1). The first criterion is the occurrence of an “interfacial activation” state, which means an increased activity of lipase during emulsion by the triglycerides [18]. The second criterion is an active site of the enzymes consisting of a surface loop “lid” that moves together with the interface [17], [22], [23]. These two criteria are important, particularly for the function-based approach.

Various prior studies [24], [25], [26] have noted the importance of lipase in the industry, the growing demand for lipases encourages more exploring for novel lipases and innovative properties of the known lipases.

## Novel lipases found in the environmental samples

In recent years, there has been growing interest in finding microbial lipases, principally from bacteria and fungi (Table 2) [27], [28], [29], [30], [31], [32], [33], [34], [35]. Interestingly, these microorganisms are very attractive as biocatalysts due to their unique properties of adapting to extreme environmental conditions such as hypersaline habitats, high pressure, and extreme temperature. In particular, some microorganisms are able to live in marine environments characterized by high levels of pollutants (*e.g*. the Norwegian Sea and the Red Sea), high pressures, high temperatures ranging 50–70 °C, little to no light or oxygen, and high concentrations of salt and heavy metals [36]. As an example of the extreme environmental conditions that are expected to enhance the activities of microbial lipases, we focus on the Red Sea and the microorganisms living there in the present review.

## Origin and history of the Red Sea

The Red Sea rift initiated the separation of the African and Arabian (Asian) continent masses about 70 million years ago [37], and the rifting took place multiple times afterward, leading to the eventual formation of the Red Sea. Moreover, frequent episodes of volcanic activities gave rise to the creation of volcanic islands in the Red Sea [37].

The Red Sea’s essential properties make it very unique in the tropics such as no river inflow to the Red Sea. Rainfall is limited between October and May. It is characterized by high salinity, which is estimated in the northern Red Sea to be approximately 40.0 practical salinity unit (psu) [38]. The temperature of surface seawater varies from 20 °C in spring to 35 °C in summer. Since one of the effects by the global warming is the increase in the seawater temperature, thus the Red Sea is attractive to researchers working on climate change. Moreover, a high amount of radiant energy exists throughout the year, reaching its peak in June [39].

The Red Sea has long been considered one of the most diverse and the warmest regions in the world [38], [40]. It is a geologically young sea basin that has experienced a conversion from a continental rift to a true oceanic seafloor, producing the high temperature seawater with high concentration of the minerals. Thus, the Red Sea is thought to be an interesting environment to study critical problems such as the microorganism adaptation to the semi-extreme environments as above [41]. In addition, these diverse environments with the adaptation can provide a quite different spectrum of microbial diversity in the Red Sea. Thus, the Red Sea can be regarded as a potential source for finding out novel enzymes of lipases.

## Challenges in conducting functional screening of marine metagenomics libraries

Recently, a considerable amount of literature has emerged on isolating lipase enzymes of microbial origin as shown in Table 2. As shown in Figure 1, DNA extracted from environmental samples can be cloned into plasmids, fosmids, bacterial artificial chromosomes, or cosmids for proliferation in a suitable heterologous host organism, such as *Escherichia coli*, and then be screened for catalytic activities. The rapid development of functional screening on metagenomic libraries to find a new enzymatic activity has indicated the importance of microbial diversity in the novel enzyme detection [42], [43], [44], [45]. However, the DNA quality of the environmental samples remains a challenge, because of the low copy number of clones and the different insert sizes of metagenomic libraries [46]. DNA purity from marine samples has also been a problem because of the complicated and multifarious nature of the marine environments and the role of co-extracted substances, such as humic acids that inhibits biochemical reactions [47].

Currently, choosing the best host system for the construction of heterologous protein and for screening in metagenomic libraries is difficult, because it depends on the nature of target protein, such as a thermo tolerance [48]. The gram-negative bacterium, *E. coli*, is the most used organism for heterologous protein construction as a well-studied model organism. Thus, the *E. coli* system is the mostly-used host for industrial protein construction, *e.g.*, *E. coli* BL21 and K12 [49]. However, several types of proteins could not be expressed in *E. coli* due to the difference of the genetic system in *E. coli* [50]. Thus using alternative bacterial hosts like *Bacillus brevis*, *B. megaterium*, and *B. subtilis* may complement the unachievable goal in *E. coli* expressing system. *B. subtilis* and other *Bacillus* strains were suggested to be the most well-known microbes for the metagenomic libraries screening and heterologous protein construction [50]. Gram-positive *Bacillus* strains have more benefits in the field of protein production and screening in metagenomic libraries for industrial applications because of absence of lipopolysaccharides in their outer membrane, since lipopolysaccharides in gram-negative bacteria are well-known endotoxin prompting macrophages [51], [52], [53].

There are other crucial challenges to be resolved before the full potential of metagenomics can be utilized. First, a huge number of clones are required to be screened as a result of the great biodiversity in the microbial ecosystem. Establishment of high-throughput screening is crucial to identify millions of positive clones in a metagenomic library in a short time. Second, the insert size is a key issue in conducting effective metagenomic screening. For example, a plasmid vector can have a short insert size (less than 10 kb). Therefore, if we use a plasmid as a vector for the library construction, the library harbors only the short length of the target fragments. For this reason, more clones are subsequently required for successful identification of positive clones, particularly when compared with metagenomic libraries constructed from fosmid vectors (the insert size is about 40 kb) [7]. Furthermore, large clusters of genes cannot be recovered with short inserts.

To overcome the limitations of a cultivation approach, several DNA-based molecular methods have been established, including the capillary-based system of cell culturing on porous hollow-fiber membranes. An analysis of 16S rRNA genes generally supplies considerable information about the species present in an environment [45], [54]. In particular, various methods can be used to screen novel lipases, including Fourier transform infrared spectroscopy (FTIR). Interestingly, this method has already been used to examine the lipolysis of different substrates (tri octanoyl glycerol and vegetable oils) [55]. Hosokawa et al introduced a high-throughput technique for functional screening of a metagenomic library, in which unique enzymes were extracted by droplet-based microfluidics [11]. In their method, a microfluidic gel micro-droplet technique was used for co-encapsulation of metagenomic clones to screen a metagenomic library based on a lipolytic activity assay [11]. Moreover, they used droplet technology coupled with fluorescent-activated cell sorting to assist the high-throughput screening of enzyme libraries with fluorogenic substrates [56]. Thus, powerful techniques such as microfluidics have become a promising tool for screening in metagenomic libraries, especially in selecting novel catalysts.

Metagenomics can be conducted easily to identify genomic segments. However, it is a tough question on how we can obtain a microorganism itself from genomic segments. When a function-based analysis is conducted, it is ideal to have individual samples of the microorganism. This is a serious problem, because most of those microorganisms are uncultivable as mentioned before. To avoid this problem, we may identify a particular coding region in nucleotide sequences that corresponds to a given functional domain of an enzyme such as lipase. Then, using genetic engineering, this functional domain can be expressed to obtain a sufficient amount of proteins in *E. coli* or yeast for biochemical assay. If we can invoke a single cell technology; however, isolation of an individual sample of the microorganism of interest is still necessary. This remains one of the biggest challenges.

In short, while metagenomics may help improve our understanding of microbial physiology, genetics, and community ecology [42], [43], [44], it can be an advanced and powerful tool for finding out a novel enzyme that is useful for biotechnology application and industrialization.

## Challenges related to DNA sequencing and bioinformatics

Over the last few decades, metagenomics has become a fundamental tool in microbial ecology, and a revolution in metagenomic studies is poised to begin, with the support of recent developments in NGS technology. Despite these facts, metagenomics still has computational challenges that need to be addressed.

In the studies of metagenomics, environmental DNA is, in the most cases, fragmented into small segments [57]. Therefore, production of millions of small reads must be reassembled *de novo* utilizing bioinformatics tools and software. However, the reassembly of these reads into contigs is still a serious computational challenge. The reconstruction of the entire genomes of microorganisms in the environmental sample remains virtually impossible at present, although continuous advances in the development of bioinformatics software and tools will have been made.

In fact, depending on the platform used, the read lengths generated from NGS platforms mostly range 75–1000 bp [58]. Short read lengths and low depth of coverage lead to the introduction of large gaps in the assembled contigs. Hence, due to the length and number of these gaps, accurate assembly of the contigs is now difficult. Therefore, regeneration of the entire gene sequences becomes extremely difficult and even impossible [59]. These challenges can be overcome through a continuous progression of high-throughput gene sequencing technologies and the establishment of methodologies used to sequence longer reads with maximal depth and efficiency. For instance, single molecule real-time sequencing (SMRT) technology, the third-generation sequencing platform, is the latest system developed by Pacific Biosciences [60]. This system has the ability to resolve such problems in current gene sequencing platforms by producing longer reads, up to 60 kb with the PacBio RS II platform (http://www.pacificbiosciences.com). Increased depth coverage and long overlapping reads allow reconstruction of a genome with fewer obstacles, in our prediction.

Instead, there is an approach to annotate the metagenome data without reassembly, *i.e.*, by classifying the NGS read directly. Functional and taxonomical classifications are the most important processes to reveal the feature of the microbial community and to find the useful enzymes. However, like the assembly of the short reads, the shortness of the NGS reads also prevents the accurate and fast classification. Several software and algorithms to solve this issue such as MetaCV [61] and CVTree3 [62] have been developed and main web resources for metagenomics studies are reviewed in this issue as well [63].

Gene prediction is also a challenge in the sequencing of metagenomics data. Many current gene finder systems require long stretches of the sequence to differentiate coding from non-coding sequences. They usually need to train sequences from a single species that is afterward utilized to generate a species-specific gene prediction model [64]. Unfortunately, this is inadequate for metagenomes that are constructed from a variety of sequences from distinct microorganisms and frequently constitute not only a limited number of long contigs but also short assemblies and unassembled reads [64]. Moreover, metagenomes are usually permeated with frameshifts that make gene prediction in metagenomes an ambitious task [65]. To overcome these issues, the bioinformatics tools to predict genes from metagenome data are actively developed such as MetaGeneMark [66], FragGeneScan [67], and MetaGeneAnnotator [68].

## Conclusion

Marine metagenomics is a fast-developing and promising area of genomic studies, by which we can investigate the microbial communities in marine environments. Marine metagenomics has already opened new avenues of research by uncovering a remarkable diversity of marine microorganisms and providing a chance of access to this microbial diversity in laboratory. Marine metagenomics can be used alternatively without culturing microorganisms to discover unique biocatalysts for new functions applied in biotechnological applications, such as lipase enzymes [4], [69]. In fact, the development of metagenomics has increased the discovery of biocatalysts as many demonstrating novel characteristics. To date, however, most biocatalysts remain uncharacterized. Biocatalysts discovery remains a challenge even with the increased functional screening capabilities.

## Competing interests

The authors have declared no competing interests.

## Figures and Tables

**Table 1:** Lipase and enzyme classification according to EC number

**EC number**	**Enzyme**
EC 1.-.-.-	Oxidoreductases
EC 2.-.-.-	Transferases
EC 3.-.-.-	Hydrolases
EC 3.1.-.-	Acting on ester bonds
EC 3.1.1.-	Carboxylic ester hydrolases
**EC 3.1.1.3**	**Triacylglycerol lipase (=lipase, in general)**
EC 3.2.-.-	Glycosylases
EC 3.3.-.-	Acting on ether bonds
EC 3.4.-.-	Acting on peptide bonds (peptide hydrolases)
EC 3.5.-.-	Acting on carbon–nitrogen bonds, other than peptide bonds
EC 3.6.-.-	Acting on acid anhydrides
EC 3.7.-.-	Acting on carbon–carbon bonds
EC 3.8.-.-	Acting on halide bonds
EC 3.9.-.-	Acting on phosphorus–nitrogen bonds
EC 3.10.-.-	Acting on sulfur–nitrogen bonds
EC 3.11.-.-	Acting on carbon−phosphorus bonds
EC 3.12.-.-	Acting on sulfur–sulfur bonds
EC 3.13.-.-	Acting on carbon–sulfur bonds
EC 4.-.-.-	Lyases
EC 5.-.-.-	Isomerases
EC 6.-.-.-	Ligases

*Note:* EC numbers and their descriptions are adapted from the Nomenclature Committee of the International Union of Biochemistry and Molecular Biology. Lipase is highlighted in bold to show its position among the EC classification.

**Table 2:** List of representative bacterial lipases

**Species**	**Refs.**
*Bacillus* sp.	[24]
*Brevibacterium* sp.	[24]
*Geobacillus thermodenitrificans*	[25]
*Gracilibacillus* sp.	[26]
*Mycobacterium tuberculosis*	[27]
*Nectria haematococca*	[28]
*Oceanobacillus* sp.	[29]
*Pseudomonas* sp.	[30], [31]
*Staphylococcus* sp.	[24]
*Virgibacillus* sp.	[29]
*Yarrowia lipolytica*	[32]
